# Icariin Ameliorates Lower Back Pain in Rats via Suppressing the Secretion of Cytokine-Induced Neutrophil Chemoatractant-1

**DOI:** 10.1155/2020/4670604

**Published:** 2020-08-05

**Authors:** Jitian Li, Manli Luo, Shaochun Wang, Guoguo Jin, Zongchang Han, Yan Ma, Guoqing Tang, Zhiping Guo

**Affiliations:** ^1^Henan Luoyang Orthopedic Hospital (Henan Provincial Orthopedic Hospital), Zhengzhou 450000, China; ^2^Henan Provincial Orthopedic Institute, Zhengzhou 450000, China; ^3^Kunshan Hospital of Traditional Chinese Medicine, Kunshan 215300, China

## Abstract

**Purpose:**

To investigate whether icariin (ICA), a well-known medicine extracted from the stem and leaf of *Epimedium brevicornum* Maxim, had analgesic effect on lower back pain (LBP) in rats.

**Methods:**

In a puncture-induced LBP rat model, the severity of LBP was quantified using the paw/foot withdrawal threshold method after intragastric administration of ICA at a dosage of 50 mg/kg/d or 100 mg/kg/d. The pain-related peptides of substance P (SP) and calcitonin gene-related peptide (CGRP) were also measured in intervertebral disc (IVD) tissue using RT-PCR after ICA treatment. In addition, the expression of cytokine-induced neutrophil chemoattractant-1 (CINC-1) in IVD was quantified using RT-PCR and ELISA examination.

**Results:**

ICA treatment resulted in a significant amelioration of mechanical allodynia in a dose-response manner, and the analgesic effect could last for two weeks even during the washout period. More importantly, the mechanism of analgesic pharmacological effect in ICA was to suppress the upregulated CINC-1, the homolog of IL-8 in rats, which is a crucial proalgesic factor contributing to LBP, in IVDs.

**Conclusion:**

ICA is a novel herbal extract to relieve LBP, and it may be a promising alternative pain killer in the future.

## 1. Introduction

Lower back pain (LBP) is a serious public health problem all over the world, and it consumes a great deal of medical resources [1]. According to the epidemiological investigation, the mean point prevalence of LBP has been estimated to be 18.3%, with a 1-month prevalence of 30.8% [2]. To meet the current and future therapeutic needs, the discovery and validation of new drug targets to effectively control LBP is a major priority for all clinicians.

The classical medicine to treat LBP is nonsteroidal anti-inflammatory drugs (NSAIDs) or opioids [3]. Although they are effective, their side effects, such as gastrointestinal mucosal injury, cardiovascular damage, and drug addiction, are obvious. In our clinical practice, we effectively alleviated acute or chronic LBP with an alternative Chinese traditional herbal recipe called Zhuchun pills. In the composition of Zhuchun pills, herb *Epimedium brevicornum* Maxim was considered the key ingredient because it was thought to have a strong association with LBD according to the theory of traditional Chinese medicine (TCM).

Icariin (ICA) is the main ingredient extracted from the stem and leaf of *Epimedium brevicornum* Maxim [4]. It was reported that ICA was able to relieve many intervertebral disc- (IVD-) related diseases. *In vitro*, ICA remarkably inhibited the IL-1*β*-induced upregulation of cyclooxygenase-2 (COX-2), nitric oxide synthase (iNOS), and prostaglandin E2 in human nucleus pulposus cells (NPCs) [5]. Alternatively, ICA was reported to reduce the apoptosis of IL-1*β*-treated NPCs as well [6]. Also *in vivo*, administration of ICA was able to ameliorate IVD degeneration in a rat model by promoting nuclear factor erythroid 2-related factor 2 (Nrf-2) activity and preserving the extracellular matrix in NPCs [7]. Hence, ICA should be the main bioactive component of *Epimedium brevicornum* Maxim to treat IVD-related disease.

With respect to pain relief, a previous study suggested that ICA played a critical role in relieving paclitaxel-induced neuropathic pain in a sirtuin-1- (SIRT1-) dependent manner [8]. However, there were no reports concerning the ability of ICA in LBP treatment. Thus, the first hypothesis that we proposed here was whether ICA had an analgesic effect in LBP treatment and whether its effect was comparable to conventional NSAIDs. In addition, the mechanism of ICA-alleviated LBP was investigated. To our knowledge, this was the first study to focus on the analgesic effect of ICA in LBP, and it may provide a new strategy in clinical practice.

## 2. Materials and Methods

### 2.1. Establishment of an LBP Model in Rats

**Figure 1:**
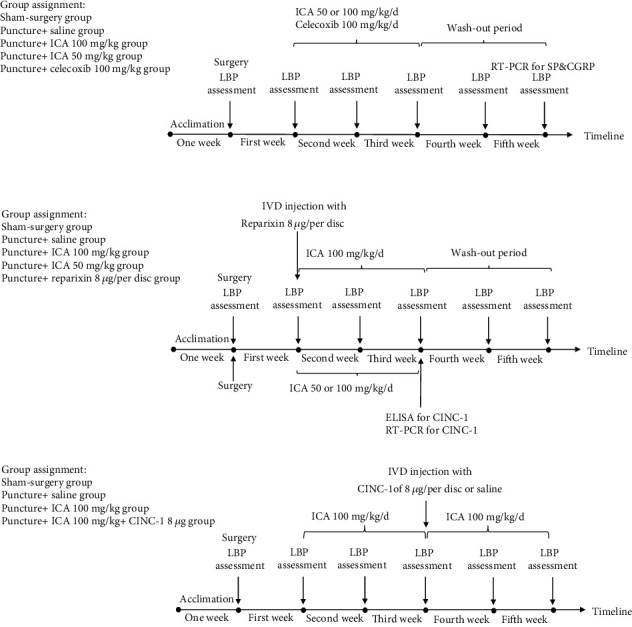
Schematic illustration of the timeline and group assignment.

Male rats weighing 250–300 g were included in this study. After anaesthetization, a transabdominal median approach was used to expose the targeted IVDs (lumbar 4-5 and lumbar 5-6) based on a previous report [9]. All animal experiments were performed in accordance with the protocol approved by Henan Provincial Orthopedic Institute of the Animal Care and Use Committee and the National Institutes of Health Guide for the Care and Use of Laboratory Animals (NIH Publications No. 8023, revised 1978). Before surgery, all animals were given one week for acclimation. An 18-gauge needle was vertically penetrated into the nucleus pulposus, and the depth of puncture was determined according to preoperative X-ray films [10]. The sham-surgery group (only exposure and no puncture), the puncture+saline group, the puncture+ICA 50 mg/kg group, the puncture+ICA 100 mg/kg group, the puncture+celecoxib group, the puncture+reparixin group, and the puncture+ICA 100 mg/kg+cytokine-induced neutrophil chemoattractant-1 (CINC-1) group were established. The author JT Li was responsible for surgery and drug delivery, while two authors who were blinded to the group assignment conducted the pain measurement. No antibiotic and analgesic drugs were used before or after the surgery. A detailed description of the timeline and group assignment is shown in Figure 1.

### 2.2. Administration of the Drug

ICA (purity≧94%, cat No. I1286, Sigma-Aldrich, MO, USA) was dissolved in deionized water, and the rats were intragastrically administered with ICA at a dosage of 50 mg/kg/d or 100 mg/kg/d. Both the two dosages were safe for rodents according to previous studies [11]. The administration of ICA began on the 7^th^ day after the surgery and ended on the 21st day. Subsequently, two weeks was observed as the washout period. In addition, celecoxib at a dosage of 100 mg/kg/d (Pfizer, Piscataway, NJ, USA) [12] was administered intragastrically in the positive control group. Reparixin (antagonist of CINC-1 receptor L-lysin salt, MedChem Express, Stockholm, Sweden) or exogenous CINC-1 (cat No.C9709, Sigma-Aldrich, MO, USA) was administered using X-ray-guided percutaneous injection at a dosage of 8 *μ*g/per disc with a 28-gauge needle. A detailed description of the timeline and group assignment is shown in Figure 1.

### 2.3. Evaluation of LBP with Paw/Foot Withdrawal Threshold in Rats

Based on a previous report, the LBP of rats was quantified using the paw/foot withdrawal threshold method [9]. Briefly, the rats were placed for acclimation at least 10 min before testing. Subsequently, calibrated von Frey filaments (Stoelting, Wood Dale, IL, USA) were applied perpendicular to the plantar surface of the hind limb with a sufficient bending force for 3–5 s. A positive reaction was defined as a brisk movement with or without lipping or biting. When the rats showed a positive reaction, a sequential lower stimulus (smaller filament) was applied. In contrast, in the event of a negative response, a greater stimulus (the next largest filament) was used. There was a one-minute interval between every two stimuli. The level of withdrawal threshold was calculated, and the average of two hind-limb scores was recorded. Assessment was conducted every week until the fifth week.

### 2.4. Real-Time Quantitative PCR

For substance P (SP), calcitonin gene-related peptide (CGRP), and CINC-1 gene expression test, the IVDs were collected from all groups. Total RNA was isolated using Trizol reagent (Invitrogen, Life Technologies Corporation, CA, USA), and cDNA was synthesized from 1 *μ*g of total RNA using reverse transcriptase (TakaRa, Shiga, Japan). An ABI 7500 Sequencing Detection System (Applied Biosystems, CA, USA) was used for the qRT-PCR process with the SYBR Premix Ex Tag Kit (TakaRa, Shiga, Japan). Cycling conditions were as follows: 40 cycles of denaturation at 95°C for 5 s and amplification at 60°C for 24 s. GAPDH served as the housekeeping gene, and all reactions were run in triplicate. The primer sequences (Sangon Biotech, Shanghai, China) used in this study were as follows:

*Rat GAPDH*: forward 5′-ATGACTCTACCCACGGCAAG-3′, reverse 5′-TACTCAGCACCAGCATCACC-3′

*Rat CINC-1*: forward 5′- AGACAGTGGCAGGGATTCAC-3′, reverse 5′-CGCGACCATTCTTGAGTGT-3′

*Rat substance P*: forward 5′-TGGTCAGATCTCTCACAAAGG-3′, reverse 5′-TGCATTGCGCTTCTTTCATA-3′

*Rat CGRP*: forward 5′-TCTAGTGTCACTGCCCAGAAGAGA-3′; reverse 5′-GGCACAAAGTTGTCCTTCACCACA-3′

The target gene expression level was normalized to the expression level of GAPDH using the 2^-*△△*^Ct method. All data were then normalized to the average values of the control group.

### 2.5. Enzyme-Linked Immunosorbent Assay (ELISA)

The rat IVD tissues were collected and homogenized in l× PBS, and then, they were centrifuged at 12,000 × g for 15 min at 4°C to collect the supernatants. The supernatants were collected after centrifugation at 10,000 × g at 4°C for 5 min. The quantification of CINC-1 was determined using a specific CINC-1 ELISA kit (R&D Systems, MN, USA). The final result of tissue samples was calculated as the CINC-1 concentration divided by the total protein concentration that was detected using the BCA assay (Beyotime Biotechnology, Shanghai, China).

### 2.6. Statistical Analysis

All data are presented as mean ± SD. For multiple group analysis, One-way or two-way ANOVA with post hoc Tukey's multiple comparison test was used with GraphPad Prism version 6. For all statistical tests, we considered a *P* value < 0.05 to be statistically significant.

## 3. Results

### 3.1. Administration of ICA Attenuated LBP in Rats

**Figure 2:**
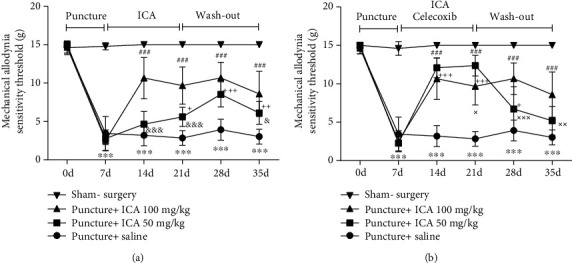
ICA treatment alleviated discogenic LBP. (a) Seven days after puncture, the animals showed obvious LBP; however, administration of ICA significantly alleviated the LBP at a dosage of 50 mg/kg/d or 100 mg/kg/d. (b) The analgesic effect of ICA treatment was similar to that of celecoxib, and the effect of ICA was more long-lasting (^∗∗∗^<0.001 on comparison between the sham-surgery group and the puncture+saline group. ^+^<0.05, ^++^<0.01, and ^+++^<0.001 on comparison between the puncture+saline group and the puncture+ICA 50 mg/kg group. ^###^<0.001 indicated the comparison between the puncture+saline group and the puncture+ICA 100 mg/kg. ^&^<0.05 and ^&&&^<0.001 on comparison between the puncture+ICA 50 mg/kg group and the puncture+ICA 100 mg/kg group. ^×^<0.05, ^××^<0.01, and ^×××^<0.001 when compared between the puncture+ICA 100 mg/kg group and the puncture+celecoxib group. The data are shown as mean ± SD. *N* = 5 for each group. Two-way ANOVA and Tukey's multiple comparison test were used for statistical analysis).

After puncture of the IVDs, all rats showed an obvious LBP indicating that the paw/foot withdrawal threshold was significantly decreased when compared to that in the sham-surgery group on the seventh day (Figure 2(a)). Then, intragastric administration of ICA significantly attenuated the LBP, indicated by a gradual increase in the paw/foot withdrawal threshold since the 14^th^ day. Although the paw/foot withdrawal threshold decreased again during the washout period, the two groups with ICA treatment still had a higher paw/foot withdrawal threshold than the saline group until the end of the observation on the 29^th^ day. In the meantime, there was a significant dose-response manner that ICA at the dosage of 100 mg/kg/d was better for LBP treatment, as depicted in Figure 2(a).

To further determine the analgesic effect of ICA, we next sought to make a comparison between ICA and celecoxib, a well-known cyclooxygenase-2 (COX-2) inhibitor as an analgesic for LBP [13]. As depicted in Figure 2(b), ICA at a dosage of 100 mg/kg/d had a similar analgesic effect as that of celecoxib at a dosage of 100 mg/kg/d, and there was no statistical difference until the 21^st^ day. However, during the washout period, the analgesic effect of ICA was better than that of celecoxib treatment with a statistical significance, suggesting that ICA treatment was more long-lasting (Figure 2(a)). Therefore, all data suggested that ICA was an effective long-lasting analgesic drug for LBP in rats.

### 3.2. Treatment with ICA Decreased the Expression of SP and CGRP in the IVDs

**Figure 3:**
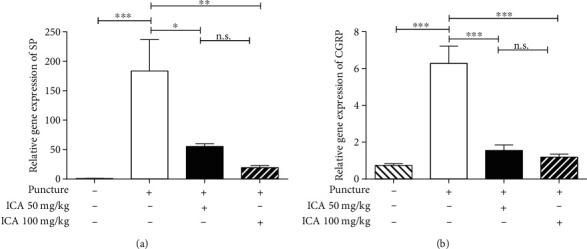
Administration of ICA suppressed the expression of pain-related peptides such as SP and CGRP. (a) At the end of observation, the expression of SP was significantly increased in the puncture+saline group when compared with the sham-surgery group; however, treatment with ICA significantly suppressed the overexpression. (b) A similar trend was seen in the expression of CGRP (^∗^<0.05, ^∗∗^<0.01, and ^∗∗∗^<0.001 when compared between each group. The data are shown as mean ± SD. *N* = 3~5 for each group. One-way ANOVA and Tukey's multiple comparison test were used for statistical analysis).

In addition to behavioral evaluation, pain-related peptides such as SP and CGRP were analyzed using RT-PCR in the IVDs. On the 29^th^ day, IVD treatment with ICA at either 50 mg/kg/d or 100 mg/kg/d caused a lower expression of SP and CGRP than that in the saline group, which objectively suggested that ICA was able to abolish the pain. However, there was no statistical difference between the ICA 50 mg/kg group and the ICA 100 mg/kg group (Figures 3(a) and 3(b)).

### 3.3. Administration of ICA Suppressed the Upregulated CINC-1 to Reduce LBP

**Figure 4:**
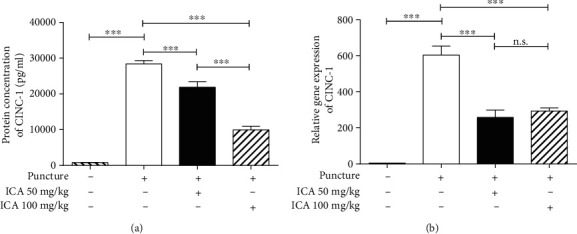
Treatment with ICA inhibited the expression of CINC-1. (a) Compared with the sham-surgery group, puncture of the disc resulted in a significant increase in CINC-1, but ICA was able to suppress the production of CINC-1 in a dose-response manner. (b) The gene expression was also decreased with ICA treatment when compared with the puncture+saline group (^∗∗∗^<0.001 when compared between each group. The data are shown as mean ± SD. *N* = 3 for each group. One-way ANOVA and Tukey's multiple comparison test were used for statistical analysis).

Many studies have demonstrated that CINC-1, the homolog of IL-8 in rats, is a crucial proalgesic factor contributing to LBP, and CINC-1/IL-8 would show significant upregulation after damage to the IVDs [9, 14]. Therefore, we tried to determine whether ICA could decrease the expression of CINC-1 and then reduce the LBP. As shown in Figure 4(a), the expression of CINC-1 protein was significantly decreased after treatment with ICA at dosages of 50 mg/kg and 100 mg/kg when compared with that in the saline injection group, and it occurred in a dose-response manner (Figure 4(a)). More importantly, the relative gene expression was also decreased by treatment with ICA at 50 mg/kg or 100 mg/kg when compared with that in the saline injection group, but it did not occur in a dose-response manner (Figure 4(b)).

**Figure 5:**
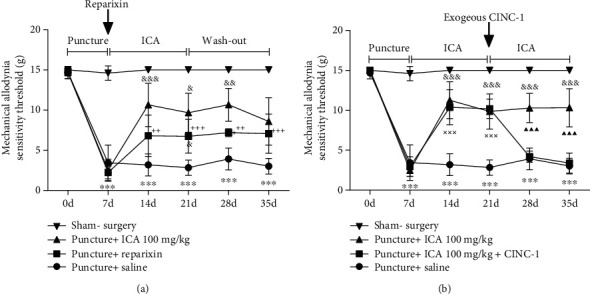
ICA attenuated the LBP by suppressing the expression of CINC-1. (a) A comparison between ICA and reparixin (the inhibitor of the CINC-1 receptor) suggested that inhibition of the production of CINC-1 was the key mechanism to attenuate discogenic LBP. (b) Percutaneous injection of exogenous CINC-1 aggravated the LBP even on treatment with ICA (^∗^<0.05, ^∗∗^<0.01, and ^∗∗∗^<0.001 when compared between the puncture+saline group and the puncture+ICA 100 mg/kg group. ^+^<0.05, ^++^<0.01, and ^+++^<0.001 when compared between the puncture+saline group and the puncture+reparixin group. ^&^<0.05, ^&&^<0.01, and ^&&&^<0.001 when compared between the puncture+ICA 100 mg/kg group and the puncture+reparixin group. ^×××^<0.001 when compared between the puncture+saline group and the puncture+ICA 100 mg/kg+CINC-1 group. ^▲▲▲^<0.001 represented the comparison between the puncture+ICA 100 mg/kg group and the puncture+ICA 100 mg/kg+CINC-1 group. The data are shown as mean ± SD. *N* = 5 for each group. One-way or two-way ANOVA and Tukey's multiple comparison test were used for statistical analysis).

Then, a behavioral evaluation suggested that ICA treatment at 100 mg/kg/d had a similar analgesic effect as that of reparixin treatment, an inhibitor of CXCR1/2, which is the primary receptor responding to CINC-1/IL-8, suggesting that inhibition of CINC-1 may be the mechanism by which ICA alleviated LBP (Figure 5(a)). Furthermore, following percutaneous injection of exogenous CINC-1, since the second week after ICA treatment, the rats showed a significantly decreased mechanical allodynia threshold, further suggesting that CINC-1 played a critical role in mediating ICA-attenuated LBP (Figure 5(b)). Therefore, it was reasonable to conclude that ICA reduced LBP via suppressing the expression of CINC-1/IL-8.

## 4. Discussion

In this study, we showed that ICA was capable of alleviating puncture-induced LBP at a dosage of 50 mg/kg/d or 100 mg/kg/d, and it occurred in a dose-response manner. Compared with celecoxib, a classical NSAID, ICA at a dosage of 100 mg/kg/d showed a similar analgesic effect but a more long-lasting effect. Finally, the pharmacological effect of ICA to attenuate LBP occurred through suppressing the upregulated CINC-1. Thus, we derived a conclusion that ICA, the main ingredient extracted from Herba Epimedii, was an effective alternative analgesic drug that acted via suppressing the upregulated CINC-1 in the IVDs.

Here, the IVDs at L4~5 and L5~6 were punctured with an 18-gauge needle based on a previous report to establish the LBP model [15]. The puncture would directly alter the mechanical properties of the IVDs, thus causing damage to the annulus fibrosus and an increase in cytokines [16], which finally caused IVD degeneration and discogenic LBP [10]. In rats, the measurement of hind-paw withdrawal threshold using mechanical stimuli was a reliable method to quantify LBP [9, 15]. Moreover, SP and CGRP, direct pain-related peptides, acted as the “biomarkers” of pain, which was in accordance with behavioral assessment, further validating LBP in rodents. Therefore, the puncture-induced LBP was a reliable model to evaluate the therapeutic effect of ICA for LBP.

For NSAIDs, the short-term analgesic effect is a problem in clinical practice. In this study, the mechanical allodynia increased again during the washout period in the celecoxib treatment group, which further validated this problem, while the ICA showed a long-term effect, as depicted in Figure 2(b). The reason for this occurrence may be that ICA suppressed the upregulation of CINC-1 since the beginning of the injury, thus inhibiting the continuous production of pain-related factors, such as SP and CGRP. Therefore, ICA had a more persistent analgesic effect than NSAIDs.

Here, we further showed that ICA was capable of alleviating puncture-induced LBP via suppressing the CINC-1/IL-8. After administration of ICA for two weeks, the level of CINC-1 was significantly decreased compared with that in the injury-only group. When compared with reparixin treatment, ICA showed similar pain relief, while injection of exogenous CINC-1 aggravated the LBP, suggesting that inhibition of CINC-1/IL-8 played an important role in treating LBP. According to accumulating evidence, increased levels of IL-8/CINC-1 in the IVDs or serum have a strong association with LBP in patients [14, 17]. In the meantime, inhibition of the effect of CINC-1 could remarkably attenuate bacterial-infected LBP [9]. The underlying mechanism was that CINC-1, the homolog of IL-18 in rat, evokes hyperalgesia via producing sympathomimetic mediators and then sensitising the nociceptors [18, 19]. For example, local pretreatment of the rats with the beta-adrenoceptor antagonist or atenolol would inhibit the CINC-1-induced hypersensitivity [18]. Hence, inhibition of CINC-1/IL-8 was one of the pharmacological mechanisms of ICA to abolish pain.

Nevertheless, there are still some limitations in this study: firstly, an *in vivo* study should be conducted to determine how ICA suppresses the upregulation of CINC-1/IL-8. In addition, the analgesic effect of ICA on chronic LBP should be observed in the future.

## 5. Conclusion

ICA is a novel effective long-lasting analgesic drug, and its pharmacological mechanism is suppression of the upregulation of CINC-1/IL-8. Considering that ICA is a natural extract of Herba Epimedii, it may be a promising alternative pain killer for LBP in the future.

## Data Availability

The datasets used and analyzed during the current study are available from the corresponding author on reasonable request.

## Abbreviations

LBP: Lower back pain
IVDs: Intervertebral discs
ICA: Icariin
NSAIDs: Nonsteroidal anti-inflammatory drugs
CINC-1: Cytokine-induced neutrophil chemoattractant-1
COX-2: Cyclooxygenase-2
NPCs: Nucleus pulposus cells.

## References

[1] Maher C., Underwood M., Buchbinder R. (2017). Non-specific low back pain. *The Lancet*.

[2] Hoy D., Bain C., Williams G. (2012). A systematic review of the global prevalence of low back pain. *Arthritis & Rheumatism*.

[3] Schreijenberg M., Koes B. W., Lin C.-W. C. (2019). Guideline recommendations on the pharmacological management of non-specific low back pain in primary care - is there a need to change?. *Expert Review of Clinical Pharmacology*.

[4] Chen S., Deng X., Ma K. (2018). Icariin improves the viability and function of cryopreserved human nucleus pulposus-derived mesenchymal stem cells. *Oxidative Medicine and Cellular Longevity*.

[5] Hua W., Zhang Y., Wu X. (2018). Icariin attenuates interleukin-1*β*-induced inflammatory response in human nucleus pulposus cells. *Current Pharmaceutical Design*.

[6] Deng X., Wu W., Liang H. (2017). Icariin prevents IL-1*β*-induced apoptosis in human nucleus pulposus via the PI3K/AKT pathway. *Evidence-based Complementary and Alternative Medicine*.

[7] Hua W., Li S., Luo R. (2020). Icariin protects human nucleus pulposus cells from hydrogen peroxide-induced mitochondria-mediated apoptosis by activating nuclear factor erythroid 2-related factor 2. *Biochimica et Biophysica Acta (BBA) - Molecular Basis of Disease*.

[8] Gui Y., Zhang J., Chen L. (2018). Icariin, a flavonoid with anti-cancer effects, alleviated paclitaxel-induced neuropathic pain in a SIRT1-dependent manner. *Molecular Pain*.

[9] Jiao Y., Lin Y., Zheng Y., Yuan Y., Chen Z., Cao P. (2019). The bacteria-positive proportion in the disc tissue samples from surgery: a systematic review and meta-analysis. *European Spine Journal*.

[10] Qian J., Ge J., Yan Q., Wu C., Yang H., Zou J. (2019). Selection of the optimal puncture needle for induction of a rat intervertebral disc degeneration model. *Pain Physician*.

[11] Wang J. L., Liu B., Zhang C. (2019). Effects of icariin on ovarian function in d-galactose-induced aging mice. *Theriogenology*.

[12] Ashkavand Z., Malekinejad H., Amniattalab A., Rezaei-Golmisheh A., Vishwanath B. S. (2012). Silymarin potentiates the anti-inflammatory effects of celecoxib on chemically induced osteoarthritis in rats. *Phytomedicine*.

[13] Kim J. S., Ahmadinia K., Li X. (2015). Development of an experimental animal model for lower back pain by percutaneous injury-induced lumbar facet joint osteoarthritis. *Journal of Cellular Physiology*.

[14] Burke J. G., Watson R. W. G., McCormack D., Dowling F. E., Walsh M. G., Fitzpatrick J. M. (2002). Intervertebral discs which cause low back pain secrete high levels of proinflammatory mediators. *The Journal of Bone and Joint Surgery British Volume*.

[15] Muralidharan A., Park T. S. W., Mackie J. T. (2017). Establishment and characterization of a novel rat model of mechanical low back pain using behavioral, pharmacologic and histologic methods. *Frontiers in Pharmacology*.

[16] Li Z., Liu H., Yang H. (2014). Both expression of cytokines and posterior annulus fibrosus rupture are essential for pain behavior changes induced by degenerative intervertebral disc: an experimental study in rats. *Journal of Orthopaedic Research*.

[17] Pedersen L. M., Schistad E., Jacobsen L. M., Roe C., Gjerstad J. (2015). Serum levels of the pro-inflammatory interleukins 6 (IL-6) and -8 (IL-8) in patients with lumbar radicular pain due to disc herniation: a 12-month prospective study. *Brain, Behavior, and Immunity*.

[18] Cunha J. M., Sachs D., Canetti C. A., Poole S., Ferreira S. H., Cunha F. Q. (2003). The critical role of leukotriene B4 in antigen-induced mechanical hyperalgesia in immunised rats. *British Journal of Pharmacology*.

[19] Lorenzetti B. B., Veiga F. H., Canetti C. A., Poole S., Cunha F. Q., Ferreira S. H. (2002). Cytokine-induced neutrophil chemoattractant 1 (CINC-1) mediates the sympathetic component of inflammatory mechanical hypersensitivitiy in rats. *European Cytokine Network*.

